# Exploring social, economic, and environmental correlates of suicide in Puerto Rico, 2017–2022: an ecological cross-sectional study

**DOI:** 10.1186/s40621-025-00632-7

**Published:** 2025-12-01

**Authors:** Alejandro Rodríguez-Putnam, Sijia He, Irina Bondarenko, Viktoryia Kalesnikava, Linh Dang, Lily Johns, Elyse Thulin, Mariluz Bezares-Salinas, Diego E. Zavala-Zegarra, Briana Mezuk

**Affiliations:** 1 Center for Social Epidemiology and Population Health, Department of Epidemiology, University of Michigan School of Public Health, Ann Arbor, MI USA; 2 Susan B. Meister Child Health Evaluation and Research Center, Department of Pediatrics, University of Michigan Medical School, Ann Arbor, MI USA; 3 Department of Biostatistics, University of Michigan School of Public Health, Ann Arbor, MI USA; 4 Institute for Firearm Injury Prevention, University of Michigan, Ann Arbor, MI USA; 5 Puerto Rico Violent Death Reporting System, Puerto Rico Institute of Statistics, San Juan, Puerto Rico; 6 Public Health Program, Ponce Health Sciences University, Ponce, Puerto Rico; 7 Research Center for Group Dynamics, Institute for Social Research, University of Michigan, Ann Arbor, MI USA

**Keywords:** Public health, Disparities, Social vulnerability, Suicide, Puerto Rico, Mental health, Cross-sectional study

## Abstract

**Background:**

Puerto Rico (PR) endures numerous natural and human-caused disasters with significant impacts on community health and infrastructure each year. The Centers for Disease Control’s Social Vulnerability Index (CDC-SVI) quantifies a community’s capacity to prepare, respond, and recover from disasters. The SVI has been linked to all-cause mortality in the continental US, however, its relevance to suicide mortality, particularly in PR, remains understudied.

**Methods:**

This cross-sectional study analyzed data from 1,471 suicide decedents recorded in the PR site of the National Violent Death Reporting System (NVDRS) from 2017 to 2022. Using Bayesian Poisson hierarchical models, we examined the associations between the county-level CDC-SVI with age- and sex-standardized suicide mortality. We explored whether contributing circumstances (i.e., mental health history, financial loss) varied by measure of SVI among these decedents.

**Results:**

During 2017–2022, there were 1,471 suicides (Mean age: 51 years, 84% male, 68% lower education) across PR’s 78 municipalities. Overall, there was no association between county CDC-SVI and suicide mortality (Relative Risk: 1.05, 95% CI: 0.88–1.25). The most common contributing circumstances (available for 93% of decedents) were mental health problems (45%), depressed mood (44%), history of mental health treatment (29%), and prior suicide attempt (28%) or suicidal thoughts (26%). Our geospatial analyses revealed a clustering of suicide and vulnerability in central counties and island-municipalities, areas marked by long-standing economic disadvantage and limited access to healthcare.

**Conclusions:**

Social vulnerability was unrelated to suicide mortality, contrasting with prior studies in the continental US. While the CDC-SVI is a useful tool for disaster response planning, its utility for suicide prevention in PR depends on the local validity and relevance of its components. These results suggest that the contextual determinants of suicide in PR operate through different structural pathways, shaped by the island’s distinct socioeconomic, demographic and disaster-exposed setting, where most communities already experience high vulnerability.

**Supplementary Information:**

The online version contains supplementary material available at 10.1186/s40621-025-00632-7.

## Introduction

Puerto Rico (PR) faces social, economic, and environmental challenges that significantly impact public health outcomes, including suicide mortality [1, 2]. Over the past two decades, PR’s demographic landscape has changed [3], largely driven by persistent economic austerity which negatively affects health-related quality of life [4, 5], accelerated population aging that is linked to high levels of outmigration [6, 7], and constant exposure to natural disasters [8, 9]. According to the United States (US) 2020 decennial Census Bureau data, there was a population decrease of more than 13% (from 3.8 million in 2000 to around 3.2 million in 2020) across all counties (municipios) in PR. Between 2005 and 2020, over 700,000 working-age individuals (ages 20–64) migrated from PR to the continental US, resulting in significant “population deficits” among younger and economically active cohorts, and leaving behind a population that is increasingly aging [7]. The decade from 2010 to 2020, which encompassed the devastating aftermath of Hurricane Maria, saw the highest number of outmigration ever recorded at 455,928 individuals [3, 10, 11]. 

Nearly half of PR’s population lives below the federal poverty level, and all 78 municipios qualify as counties of persistent poverty [12] (median household income of USD 24,036, compared to continental US’s USD 75,358) [13]. Poverty is not equally distributed in PR; there is substantial geographic variation, with rural and central counties disproportionately affected, while the highest incomes are concentrated in the San Juan metropolitan area [12, 14]. Historically, economic strife and fiscal crises have been a major driver of outmigration, with scholars emphasizing that PR’s current migration wave is linked to longstanding economic turmoil [4, 15, 16]. Natural disasters have also played a critical role in shaping the public health of PR residents, compounding these existing challenges [8, 9, 17, 18]. The devastation from Hurricane Maria in 2017, multiple earthquakes and the COVID-19 pandemic have significantly impacted the island’s health and social infrastructure and mental health of its residents [8, 9, 17, 19–22]. Hurricanes and other disasters are linked to increased psychological distress and worsened mental health outcomes, such as post-traumatic stress disorder, depression, and suicide [23–27]. Limited access to healthcare and mental health care services, as well as physician shortages, exacerbate existing vulnerabilities, particularly among rural communities [28–31]. While multiple studies have examined and documented the excess mortality in PR following hurricane Maria [32–34], there remains limited research examining mental health and suicide within this context [35–40].

Suicide remains a significant public health concern in PR, ranking as the third cause of violent death, and reflecting both social and individual-level risk factors [2, 41]. Notably, following Hurricane Maria, an increase in suicides was observed in 2017 compared to 2016 [19, 42], drawing attention to the potential interplay between disasters and suicide risk. Prior epidemiologic research of suicide mortality in PR analyzing data from 2000 to 2006 found that the highest burden was among younger male populations [43], however more recent data suggests a shift toward older adult men [41, 42].

In addition to individual-level determinants such as age and sex, broader area-level factors such as socioeconomic status, access to health and social services, or urbanicity may shape suicide risk. A neighborhood’s “social vulnerability” is defined by a combination of socioeconomic and demographic factors, and reflects both the capacity of a system to respond to an impact as well as an intrinsic lack of capability of individuals to cope with external stressors [44]. It shapes how communities withstand and recover from disasters and economic crises [45]. Disasters have disproportionate impacts across neighborhoods, with more vulnerable populations experiencing greater unmet needs, delayed access to care, and persistent health disparities in the aftermath [46]. Socially vulnerable communities are more likely to experience higher rates of mortality, morbidity and property destruction, and are less likely to fully recover from a disaster compared to communities that are less socially vulnerable [44]. The Centers for Disease Control and Prevention’s Social Vulnerability Index (CDC-SVI) [47] provides a composite measure of vulnerability based on 16 factors such as poverty, unemployment, housing conditions, and access to healthcare. The CDC-SVI has important implications for policy, guiding the allocation of federal funding for disaster preparedness and response in the US, including in PR [48].

Understanding how social vulnerability correlates with suicide risk in PR is crucial for developing targeted public health interventions. However, previous studies have found mixed evidence regarding the validity of the CDC-SVI in the contexts of post-disaster population loss [49] and critical infrastructure resiliency in PR [50]. For instance, a study of population changes after Hurricane Maria found that while the standard CDC-SVI significantly predicted population loss, a refined 10-variable version of the SVI (i.e., SVI-10) exhibited greater construct validity and stronger predictive power for local contexts [49]. Similarly, studies examining power infrastructure vulnerability [50, 51] and the impact of the COVID-19 pandemic [52] also emphasized the importance of tailoring SVI measures to the unique local demographic and socioeconomic characteristics of PR. Finally, while the US Department of Health and Human Services has recently developed the Minority Health SVI (MHSVI) to better capture risk for disproportionate impact and adverse outcomes of public health emergencies among racial/ethnic minority populations, PR was notably excluded from this index [53].

The interplay between broader societal stressors, such as long-standing economic instability and exposure to recurring disasters, with individual-level circumstances surrounding suicide remains understudied in PR. Therefore, the purpose of this study is twofold: First, to investigate the relationship between county-level social vulnerability and suicide in PR using two measures: the US-wide CDC-SVI and a PR-tailored version, the SVI-10; second, to examine the key individual-level demographic characteristics and contributing circumstances, such as alcohol or drug abuse, job or financial problems, among others, that may vary with area-level social vulnerability.

## Methods

### Study design and analytic sample selection

This ecological cross-sectional study used restricted-access data from the CDC’s National Violent Death Reporting System (NVDRS) for 2017–2022. The NVDRS collects and links primary investigative information from a number of existing sources, including death certificates (DC), coroner and medical examiner (C/ME) reports, toxicology and autopsy records, and law enforcement (LE) reports, to create the most comprehensive surveillance system of violent deaths in the US [54]. As of 2019, the NVDRS captures all 50 states, the District of Columbia, and PR, although states’ participation varies by year [54]. The PR site began reporting data to the NVDRS in 2017. The methodology of the NVDRS has been described in detail elsewhere [55].

**Fig. 1:**
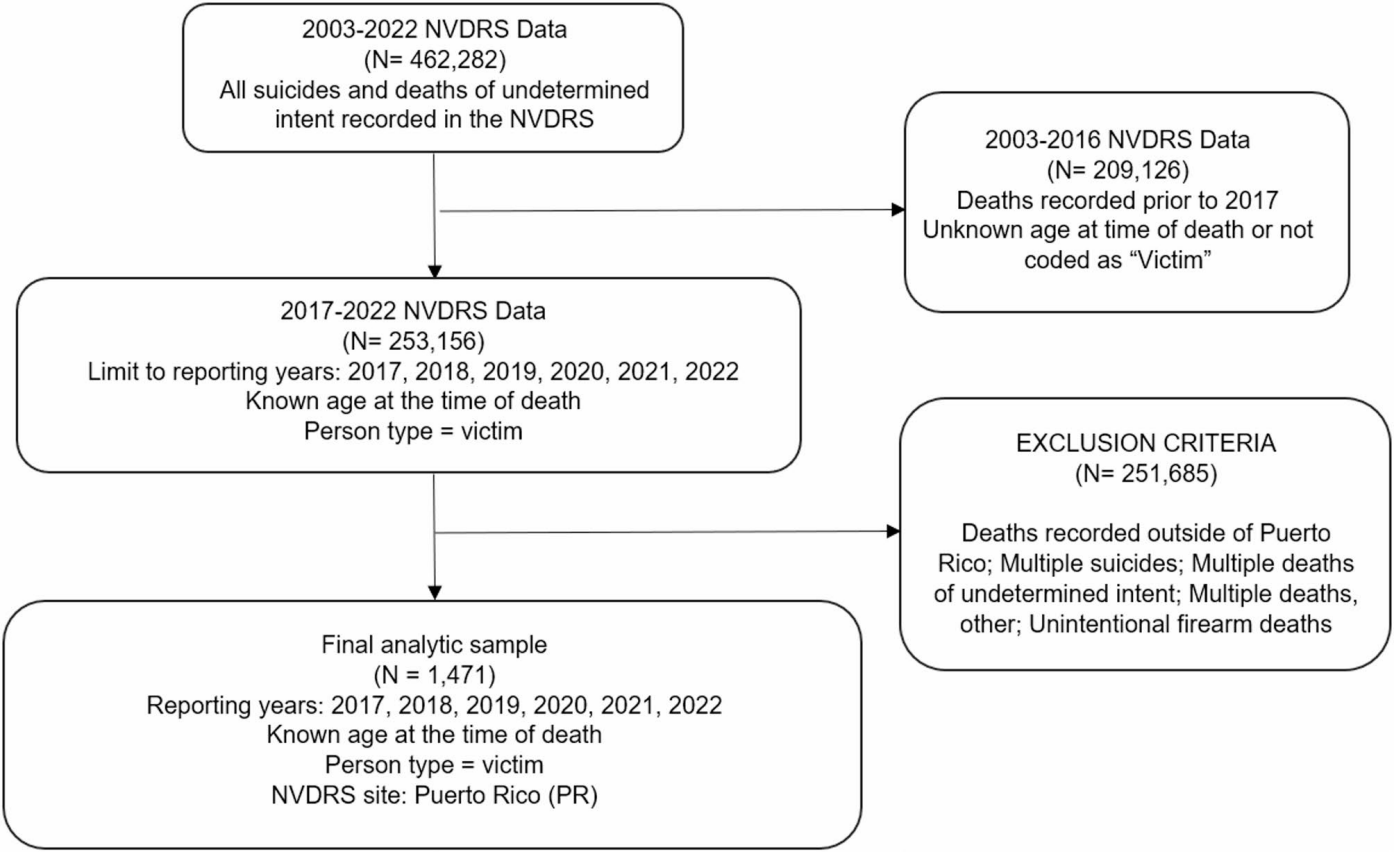
Flowchart of Analytic sample selection from the National Violent Death Reporting System. The flowchart illustrates the criteria used to generate the final analytic sample for this study. Starting with all suicides and undetermined deaths recorded in the NVDRS from 2003-2022 (N = 462,282), the sample was restricted to records from 2017-2022 with known age at time of death and decedents identified as victims (N = 253,156). Cases recorded prior to 2017, with unknown age, or not coded as victims (N= 209,126) were excluded. Additional exclusion criteria included deaths recorded outside of Puerto Rico (PR), multiple suicides, multiple deaths of undetermined intent, multiple homicides followed by suicide, unintentional firearm deaths (N=251,685). Most exclusions (>99%) corresponded to deaths recorded in other sites of the NVDRS. The final analytic sample included 1,471 suicide and deaths of undetermined intent occurring in PR during 2017-2022

The analytic sample included all single suicides (*N* = 1,389) and deaths of undetermined intent (*N* = 82) among decedents with known age occurring in PR between 2017 and 2022. We included deaths of undetermined intent because certain manners of suicide (e.g., poisoning) are often misclassified as undetermined intent, particularly among decedents with low socioeconomic status [56]. In accordance with CDC and NVDRS reporting guidelines, cases under age 10 were not reported separately because there were fewer than five during the study period; these cases were combined in the 0–24 age category in descriptive tables and figures. There was no missing data for the outcome, as all suicide deaths during the study period were recorded in the NVDRS. The final analytic sample included 1,471 decedents with known age at time of death. The sample selection process is presented in Fig. 1.

The restricted-access NVDRS data with county-level geographic identifiers and permission to link data across data sources was approved by the CDC. This study used de-identified data and was deemed exempt from human subjects regulations (HUM00209833).

### Measures

#### Outcome: county-level suicide counts and age- and sex-standardized suicide mortality ratios (SMRs)

 The primary outcome for this study was county-level suicide counts and age- and sex-standardized suicide mortality ratios (SMRs) at the county level in PR from 2017 to 2022. Suicide deaths were identified in the NVDRS, using the International Classification of Diseases, Tenth Revision, Clinical Modification (ICD-10-CM) codes for suicide (X60-X84, Y87.0, and U03) and undetermined death (Y10-Y34, Y87.2, and Y89.9). We calculated SMRs using indirect standardization by dividing the observed number of suicide deaths in each county by the expected number, which was based on the county’s age and sex composition and calculated using PR’s overall age- and sex-specific suicide rates for 2017–2022 (see Additional File 1 for formula). County-level population estimates for age and sex were obtained from the 2018–2022 Puerto Rico Community Survey (PRCS) 5-year estimates [57]. Direct standardization was not feasible due to sparse data and small population sizes in several counties. Therefore, we modeled observed counts with expected counts as offsets in Bayesian Poisson spatial models to stabilize estimates, an approach recommended for rare outcomes [58].

##### Decedent characteristics

We included the following NVDRS quantitative variables: age group [coded as 0–24, 25–44, 45–64 and 65 and older]; sex [male, female]; race/ethnicity [coded as white hispanic/latino, black hispanic/latino, other/unknown]; education [coded as high school or less (i.e., <= 8th grade, 9-12th grade, HS/GE) and college or more (i.e., associate’s, bachelor’s, master’s, doctorate degree)]; marital status [coded as married, widowed/divorced/separated, single/never married]; autopsy status [full/partial vs. no autopsy]; and means of injury [firearm, poison, hanging/suffocation, sharp/blunt instrument, fall, other or unknown].

##### Contributing circumstances

The NVDRS provides detailed information about the circumstances leading up to each death, based on C/ME, LE, and DC reports. Although measures of community vulnerability provide important context, studies have shown that individual-level circumstances preceding suicide, such as those related to traumatic events or substance use, can exhibit distinct geographic clustering that may not always align with area-level indicators [59]. Our analysis used the quantitative “circumstance variables” in the NVDRS which capture precipitating factors, events or conditions occurring prior to the fatal injury, as identified in any part of the investigative record.

We created a series of binary variables (“Yes” versus “No/Not available/Unknown”) to indicate the presence or absence of key circumstances, including: mental health problem, depressed mood, current mental health treatment, history of mental health treatment, previous suicide attempt, previous suicidal thoughts, alcohol problem, substance abuse, physical health problem, recent death of family or friend, job or financial problem, any recent crisis, recent argument, intimate partner problem, family relationship problem and disaster exposure. The NVDRS combined “No” and “Unknown” in the same category due to the challenges in determining if the circumstance did not occur or occurred but was not present in investigative reports.

##### Primary exposure: CDC-social vulnerability index

The CDC-SVI was constructed at county level using the 2018–2022 PRCS, which is adapted from the American Community Survey (ACS) to account for cultural and geographic differences between PR and the continental US [60]. Counties serve as meaningful units of analyses for assessing social vulnerability because they function as independent legal and political entities, with distinct economic budgets, social services, and infrastructure. The CDC-SVI facilitates the comparisons of relative social vulnerability between different geographic areas for emergency preparedness and public health response by ranking counties across 16 vulnerability indicators in four core themes:

1. **Socioeconomic Status: **Percentage of persons living below 150% of the poverty level, unemployment rate, housing cost burden, percentage of persons age ≥25 without a high school diploma, percentage of persons without health insurance.
2. **Household Composition and Disability: **Percentage of persons aged ≥65, percentage of persons aged ≤17, percentage of persons with a disability, percentage of single-parent households with children under 18, percentage of households with limited English proficiency.
3. **Racial & Ethnic Minority Status: **Percentage of minority (Hispanic or Latino, Black or African American, American Indian and Alaska Native, Native Hawaiian and other Pacific Islander, two or more races).
4. **Housing type and Transportation: **Percentage of housing units in multi-unit structures, percentage of mobile homes, percentage of households with more occupants than rooms (crowding), percentage of households without vehicle access, percentage of persons living in group quarters.

The CDC-SVI rankings were based on percentiles; percentile ranking values ranging from 0 (least vulnerable) to 1 (most vulnerable), with higher values indicating greater social vulnerability [47]. For each county, its percentile rank was calculated for 16 individual vulnerability indicators, the four themes, and the overall score. The theme-specific percentile rankings were constructed by summing the percentiles for the indicators comprising a particular theme and ranking the summed percentiles for each theme. These theme-specific SVIs reflect the social vulnerability within specific domains, allowing for more targeted disaster response efforts. Finally, to provide a single summary measure of overall vulnerability, the overall composite SVI was constructed by summing the percentiles across four themes and ranking the summed percentiles across all counties [47]. In the present analysis, counties were assigned to SVI tertiles (Low, Medium, High) based on the overall composite percentile, with cut points based on rank ordering across all 78 counties. We linked data across sources (e.g., NVDRS and CDC-SVI) using county Federal Information Processing Standards (FIPS) codes in the CDC-SVI and the “ResidenceFIPS” variable from NVDRS.

##### Application of the PR-adapted SVI-10

As articulated by West [49], the CDC-SVI includes several elements that are less-relevant and/or not applicable to the PR context (e.g., English language proficiency, given that nearly all PR residents speak Spanish as a first language). Therefore, as a sensitivity analysis, we implemented a 10-item version of the SVI (SVI-10) which tailors the CDC-SVI to the context of PR by applying the methodology previously developed by West [49].

Using exploratory factor analysis (EFA) with varimax rotation on 16 vulnerability indicators from the 2018–2022 CDC-SVI, we retained indicators with strong factor loadings (≥ 0.40), acceptable communalities, and clear theoretical relevance to PR’s context. Factor loadings represent the correlation between an observed variable and an underlying latent factor. Consistent with West’s findings, we excluded mobile homes and group quarters due to poor statistical performance (e.g., uniqueness values of 0.95 and 0.89 respectively and communalities < 0.11, suggesting that they did not meaningfully load onto any latent factor). Multi-unit housing showed an inverse loading direction relative to vulnerability and lacked conceptual alignment with other socioeconomic risk factors, thus was excluded. We also excluded no vehicle access because it exhibited high complexity, indicating inconsistent relationships with latent social vulnerability constructs. In addition, racial/ethnic minority status and limited English proficiency were excluded on theoretical grounds, given that 99% of the PR population identifies as Hispanic or Latino and Spanish is the primary language spoken on the island. A table with the SVI variables retained and excluded from CDC-SVI and the SVI-10 is available in Supplementary Table 1, Additional File 2. Further details on EFA are also presented in Supplementary Tables 2 and Supplementary Fig. 1, Additional File 2.

### Analytic approach

#### Descriptive and Geospatial analyses

First, the sociodemographic profile of suicide decedents in PR from 2017 to 2022 by CDC-SVI tertiles was compared using ANOVA for continuous variables and Pearson’s Chi-squared test for categorical variables. In addition, contributing circumstances among PR’s suicide decedents were characterized by CDC-SVI tertiles. To aggregate individual-level data by county and stratify by social vulnerability, counties were grouped into low, medium, and high vulnerability tertiles based on county-level CDC-SVI values, and each decedent was assigned the SVI tertile corresponding to their county of residence.

Then, to illustrate spatial variation and clustering, we mapped SMRs and social vulnerability indexes (CDC-SVI and SVI-10) across all 78 counties in PR. County boundaries were derived from publicly available shapefiles and linked via FIPS codes.

#### Multilevel modeling of the association between social vulnerability and suicide mortality

The association between county-level social vulnerability and suicide mortality was evaluated using Bayesian multilevel Poisson regression models. Bayesian spatial models are an effective way to examine rare outcomes such as suicide deaths, where sparse data at the county level can lead to unstable estimates [61]. These models produce smoothed estimates of relative risk (RR) by utilizing partial pooling, in which each county-specific risk was estimated not only directly from suicide deaths in that particular county but also indirectly from observations of its neighboring counties, thus generating more stable relative risk estimates, especially for counties with sparse data.

 To address spatial autocorrelation and overdispersion among neighboring counties, we modeled the observed counts of suicide deaths at the county level, including the expected number of deaths (derived from PR’s age- and sex-specific rates) as an offset term. We then specified a spatially explicit multi-level framework. We specified a modified Besag-York-Mollié type 2 (BYM2) random effects structure that models both structured spatial variation (based on county adjacency) and unstructured heterogeneity (random intercepts for overdispersion) [62]. The spatial neighborhood matrix was constructed using contiguity-based adjacency (shared borders). The Integrated Nested Laplace Approximation (INLA) approach, with penalized complexity priors for random effect precision and spatial structure, were used for Bayesian estimation of latent Gaussian models [63]. Model fit was assessed using the deviance information criterion (DIC) and Watanabe-Akaike information criteria (WIC), with lower values indicating improved fit.

In all analyses, the observed count of suicide deaths in each county was modeled as the response variable, with the expected number of deaths (derived from PR’s age- and sex-specific suicide rates and each county’s population structure) included as an offset term in the model rates. We examined two main exposures, including CDC-SVI and SVI-10, each modeled as categorical tertiles. All models were adjusted for covariates relating to healthcare access and spatial variations across counties, including population density (per US Census estimates), county-level median distance by road to the nearest emergency department, medical/surgical intensive care unit, and hospitals with inpatient alcohol and drug abuse treatment. Results are presented as RRs with 95% credible intervals (CI).

#### Sensitivity and supplementary analyses

In supplementary analyses, we fit four alternative models to evaluate robustness of our results. To explore how specific aspects of SVIs relate to suicide mortality, additional models were fit using theme-specific CDC-SVI measures (socioeconomic status, household composition and disability, racial & ethnic minority status, and housing type and transportation) as independent predictors. For analyses using SVI-10 themes, the racial & ethnic minority status theme was not examined given that this domain was excluded from the construction of SVI-10. In addition, a set of analyses, with the overall CDC-SVI and SVI-10 as exposures, using alternative model specifications were conducted. These included BYM2 models unadjusted for covariates, as well as adjusted models using alternative specifications for random effects (only random intercepts for counties, Besag model with a structured spatial random effect, and an original BYM model with random intercepts for counties and a structured spatial random effect).

Analyses were conducted in the R environment, version 2024.12.1 + 563. Significance tests were evaluated two-sided at *p* < 0.05. Spatial data processing and visualization were performed using R packages *sf*, *tigris*, and *ggplot2* (versions 1.0.19, 2.1, 3.5.1). Spatial mixed effects models were fitted using the INLA package [63] (version 24.12.11) in R version 4.4.3.

## Results

Table 1 shows the county-level characteristics by CDC-SVI tertiles, which provides a contextualized interpretation of how social vulnerability related to suicide mortality at the county level in PR. The top part of the table shows contextual characteristics that are not directly part of the creation of the SVI, and the lower part shows the component themes of the CDC-SVI. Across SVI tertiles, there were no significant differences in population density, median age, educational attainment, or median distance to healthcare facilities (i.e., emergency department, medical/surgical ICU, hospital with alcohol and drug abuse inpatient care). However, median household income was significantly lower in counties with higher social vulnerability (mean = $17,480 in the high-SVI group vs. $21,235 in the low-SVI group, *p* = 0.008).

**Table 1:** County-level characteristics by CDC-Social vulnerability index tertiles in Puerto Rico

**Characteristics (mean ± SD)**	CDC-Social vulnerability index			**p-value**
	**Low** (*N* = 26 counties)	**Medium** (*N* = 26 counties)	**High** (*N* = 26 counties)	
Population density	1154.2 ± 849.7	1053.4 ± 1017.1	1206.9 ± 1537.9	0.8
Median age	41.1 ± 1.9	40.9 ± 2.3	40.5 ± 1.8	0.5
Median household income (in USD)	21235 ± 5883	18156 ± 3992	17480 ± 2591	**0.008**
% has only high school diploma	28.7 ± 4.6	29.5 ± 4.3	31.7 ± 5.2	0.06
Median distance to nearest emergency department	5.37 ± 4.04	5.76 ± 2.83	5.66 ± 4.41	0.93
Median distance to nearest medical/surgical ICU	5.53 ± 4.04	6.38 ± 3.39	6.72 ± 5	0.57
Median distance to nearest hospital with alcohol and drug abuse inpatient care	17.57 ± 12.5	14.25 ± 8.43	16.35 ± 9.55	0.50
**16 SVI indicators**				
**SVI Theme 1: Socioeconomic status**				
% Below Poverty	58.5 ± 9.1	65.8 ± 7.8	66.4 ± 7.5	**0.001**
% Unemployed	9.2 ± 4.3	13.5 ± 4.9	17.7 ± 6.1	**< 0.0001**
% High Housing Cost Burden	20.1 ± 4.2	20.2 ± 4.3	21.9 ± 3.7	0.2
% No High School Diploma	22.0 ± 6.3	23.3 ± 5.0	23.9 ± 4.3	0.4
% Uninsured	5.6 ± 2.0	5.5 ± 2.0	6.7 ± 2.0	0.08
**SVI Theme 2: Household characteristics**				
% Aged < 18	17.2 ± 1.2	17.7 ± 1.3	18.1 ± 1.0	**0.03**
% Aged 65+	21.5 ± 2.5	21.9 ± 2.8	21.9 ± 2.2	0.8
% with Disability	22.1 ± 8.1	20.2 ± 6.5	23.1 ± 4.1	0.3
% Single-Parent Households	7.1 ± 1.5	7.8 ± 2.0	8.6 ± 2.1	**0.02**
% Limited English Proficiency	62.8 ± 9.1	66.7 ± 8.3	68.1 ± 8.8	0.08
**SVI Theme 3: Racial & ethnic minority status**				
% Minority	98.8 ± 1.4	99.4 ± 0.7	99.3 ± 0.8	0.07
**SVI Theme 4: Housing type and transportation**				
% Multi-Unit Housing	3.6 ± 4.9	3.4 ± 4.1	5.6 ± 7.1	0.3
% Mobile Homes	0.2 ± 0.2	0.4 ± 0.7	0.3 ± 0.2	0.3
% Crowded Housing	2.2 ± 1.9	2.7 ± 1.3	3.2 ± 1.5	0.08
% No Vehicle	11.6 ± 3.3	12.9 ± 2.4	15.1 ± 3.4	**0.0004**
% Living in Group Quarters	0.5 ± 0.3	0.6 ± 0.6	1.0 ± 1.4	0.08

Bold p-values indicate statistical significance (p < 0.05)

^1^p-values were calculated using Pearson’s Chi-squared test for categorical variables and ANOVA for continuous variables

^2^Social Determinants of Health Database. Agency for Healthcare Research and Quality, Rockville, MD. Available from: https://www.ahrq.gov/sdoh/data-analytics/sdoh-data.html

^3^Agency for Toxic Substances and Disease Registry. CDC SVI. 2024. US Centers for Disease Control and Prevention, Agency for Toxic Substances and Disease Registry. CDC SVI Data & Documentation Download. Available from: https://www.atsdr.cdc.gov/place-health/php/svi/svi-data-documentation-download.html

### Decedent characteristics and contributing circumstances

Table 2 shows the demographic characteristics of suicide decedents by CDC-SVI tertiles in PR. Annual suicide deaths declined over the study period, reaching their highest level in 2017 (*N* = 305) and declining in 2022 (*N* = 191). The mean expected number of suicide deaths in the decedents’ counties of residence increased across CDC-SVI tertiles, from 24.4 (standard deviation (SD) = 15.0) in low-SVI counties to 34.0 (SD = 27.1) in medium-SVI counties and 53.4 (SD = 56.9) in high-SVI counties (*p* < 0.001). Most suicide decedents were aged 45 or older (63%), male (84%), and had a high school education or less (68%). Across all tertiles, hanging or strangulation was the most common means of injury (59–63%), followed by firearm (12–14%) and poisoning (10–13%). There were no statistically significant differences in individual-level sociodemographic characteristics across the strata of CDC-SVI, including those most closely related to the SVI component indicators such as marital status or education.

**Table 2:** Demographic characteristics of suicide decedents by CDC-Social vulnerability index tertiles: Puerto Rico violent death reporting System, 2017–2022 (*N* = 1,471)^1^

**Characteristics**	CDC-Social vulnerability index			**p-value**^2^
	**Low** (*N* = 472 decedents)	**Medium** (*N* = 454 decedents)	**High** (*N* = 545 decedents)	
**Expected suicide deaths**,** mean ± SD**	24.4 ± 15.0	34.0 ± 27.1	53.4 ± 56.9	< 0.001
**Number of suicide deaths by year**,** N (%)**				0.15
2017	97 (20.6)	108 (23.8)	100 (18.3)	
2018	97 (20.6)	82 (18.1)	109 (20.0)	
2019	70 (14.9)	88 (19.4)	85 (15.6)	
2020	68 (14.4)	61 (13.4)	88 (16.1)	
2021	80 (16.9)	54 (11.9)	93 (17.1)	
2022	60 (12.7)	61 (13.4)	70 (12.8)	
**Age group**,** N (%)**				0.30
0–24^3^	36 (7.6)	39 (8.6)	41 (7.5)	
25–44	144 (31.0)	129 (28.0)	161 (30.0)	
45–64	170 (36.0)	150 (33.0)	214 (39.0)	
65+	122 (26.0)	136 (30.0)	129 (24.0)	
**Female**,** N (%)**	80 (17.0)	68 (15.0)	93 (17.0)	0.62
**Hispanic/Latino**,** N (%)**	448 (95.0)	439 (97.0)	518 (95.0)	0.30
White, Hispanic/Latino	389 (82.4)	385 (84.8)	449 (82.4)	
Black, Hispanic/Latino	40 (8.5)	44 (9.7)	46 (8.4)	
Other/Unknown	43 (9.1)	25 (5.5)	50 (9.2)	
**Education**,** N (%)**				0.57
High school or less	320 (68.0)	328 (72.0)	346 (63.0)	
College or more	139 (29.0)	112 (25.0)	180 (33.0)	
Unknown/Missing	13 (2.8)	14 (3.1)	19 (3.5)	
**Marital status**,** N (%)**				0.25
Single/Never married	215 (46.0)	198 (44.0)	256 (47.0)	
Married (excluding partnership)	115 (24.0)	114 (25.0)	122 (22.0)	
Divorced/Separated	116 (25.0)	97 (21.0)	125 (23.0)	
Widowed	16 (3.4)	34 (7.5)	28 (5.1)	
Unknown/Missing	6 (1.5)	16 (2.9)	14 (2.6)	
**Means of Injury**,** N (%)**				0.11
Hanging/Strangulation	296 (62.7)	282 (62.1)	324 (59.4)	
Firearm	66 (14.0)	64 (14.1)	68 (12.5)	
Poisoning	45 (9.5)	44 (9.7)	69 (12.7)	
Fall	14 (3.0)	21 (4.6)	40 (7.3)	
Other, Unknown	51 (10.8)	43 (9.5)	44 (8.1)	

^1^CDC-SVI tertiles are based on percentile rankings of composite scores across municipalities: Low (0.00–0.33), Medium (0.34–0.66), High (0.67–1.00)

^2^p-values were calculated using Pearson’s Chi-squared test for categorical variables and ANOVA for continuous variables

^3^Per CDC and NVDRS reporting standards, cell counts fewer than five are not reported separately to protect confidentiality. During the study period, fewer than five suicides or undetermined deaths occurred among children under age 10; these cases were retained in the analytic sample and grouped with the 0–24 age category

Table 3 shows that approximately 93% of decedents had available data on death circumstances. The most commonly reported mental health-related circumstances across CDC-SVI tertiles were any mental health problem (41–48%), depressed mood (42–47%), history of mental health treatment (25−32%), and prior suicide attempts (26−30%) or suicidal thoughts (24–27%). Mental health-related circumstances did not vary across CDC-SVI tertiles. Socioeconomic and other health-related circumstances were less common, and also showed minimal variation across tertiles. For example, job or financial problems were reported in 7–9% of decedents. Physical health problems (14–17%), alcohol problems (13–14%), and substance abuse (15–17%) had similar distribution across tertiles. Other social and relationship factors, such as recent arguments (5–6%), intimate partner problem (11–12%) and family relationship problem (2–3%) were also uncommon. Finally, exposure to a disaster, and any recent crisis were only reported in a small fraction of cases (disaster exposure: 1–4%; any recent crisis: 6–9%), with no statistically significant differences recorded by CDC-SVI tertile.

**Table 3:** Circumstances among suicide decedents by CDC-Social vulnerability index tertiles: Puerto Rico violent death reporting System, 2017–2022 (*N* = 1,471)^1^

**Circumstances**,** N (%)**	CDC-Social vulnerability index		
	**Low** (*N* = 472 decedents)	**Medium** (*N* = 454 decedents)	**High** (*N* = 545 decedents)
Circumstances Known	395 (84%)	390 (86%)	469 (86%)
Any Mental Health Problem	215 (46%)	187 (41%)	260 (48%)
Depressed Mood	209 (44%)	212 (47%)	231 (42%)
Current Mental Health Treatment	95 (20%)	91 (20%)	125 (23%)
History of Mental Health Treatment	141 (30%)	113 (25%)	172 (32%)
Previous Suicide Attempt	134 (28%)	117 (26%)	166 (30%)
Previous Suicidal Thoughts	111 (24%)	127 (28%)	150 (27%)
Alcohol Problem	62 (13%)	65 (14%)	74 (14%)
Substance Abuse	70 (15%)	69 (15%)	93 (17%)
Physical Health Problem	78 (17%)	78 (17%)	74 (14%)
Job or Financial Problem	34 (7%)	38 (8%)	50 (9%)
Any Recent Crisis^2^	38 (8%)	42 (9%)	35 (6%)
Recent Argument	30 (6%)	22 (5%)	28 (5%)
Intimate Partner Problem	54 (11%)	53 (12%)	58 (11%)
Family Relationship Problem	13 (3%)	11 (2%)	14 (3%)
Recent Death of Family or Friend	17 (4%)	23 (5%)	28 (5%)
Disaster Exposure	6 (1%)	18 (4%)	13 (2%)

^1^CDC-SVI tertiles are based on percentile rankings of composite scores across municipalities: Low (0.00–0.33), Medium (0.34–0.66), High (0.67–1.0)

^2^The “Any recent crisis” includes the following NVDRS variables: financial, job, eviction, physical health, bereavement, interpersonal, substance use, mental health, legal, school-related, and disaster-related crises, as documented in the NVDRS Coding Manual. “Crisis” is defined as a current/acute event (within 2 weeks of death) that is indicated to have contributed to the death

### Geospatial patterns

**Fig. 2:**
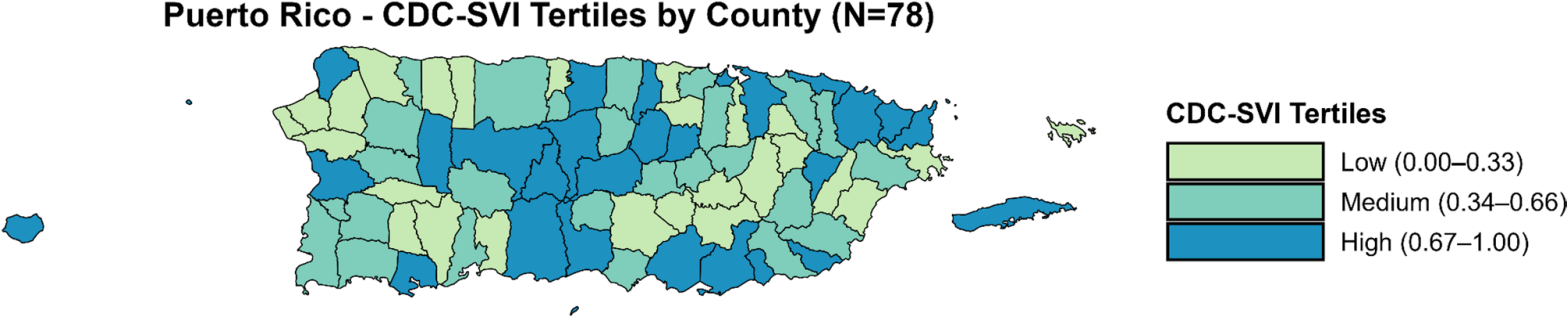
Map of County-level CDC Social Vulnerability Index (CDC-SVI) tertiles in Puerto Rico, 2018–2022 (*N* = 78). Counties are categorized into tertiles based on the CDC Social Vulnerability Index (CDC-SVI) scores for Puerto Rico: Low (0.00–0.33), Medium (0.34–0.66), High (0.67–1.0). Darker shading indicates greater social vulnerability. Tertiles are based on the distribution of SVI scores among PR’s 78 counties for the 2018-2022 period

**Fig. 3:**
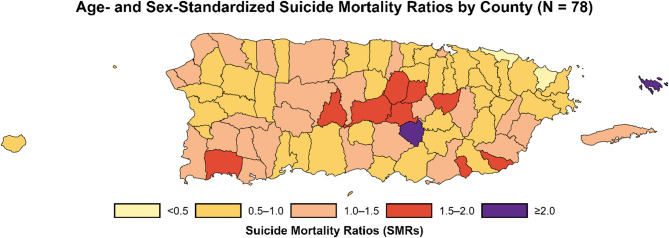
Map of Age- and sex-standardized Suicide Mortality Ratios (SMRs) by county in Puerto Rico, 2017–2022 (*N* = 78). Map of Puerto Rico’s 78 counties shaded by Age- and sex-standardized suicide mortality ratios (SMRs) from 2017 to 2022. Counties with SMRs below 1.0 (lighter shades) indicate lower-than-expected suicide mortality; while counties above 1.0 (darker shades) indicate higher-than expected mortality relative to the island-wide average. SMRs were calculated using indirect standardization based on age and sex distributions, which were obtained from the 2018-2022 Puerto Rico Community Survey (PRCS) 5-year estimates

Figure 2 and Fig.3 illustrate the spatial patterns of CDC-SVI (categorized in tertiles in Fig. 2) suicide mortality (represented as SMRs in Fig. 3) across PR’s 78 counties. As shown in Fig. 3, there were regional variations in suicide mortality from 2017 to 2022. Higher suicide rates are observed in central mountainous counties, including Aibonito (SMR = 2.33), Orocovis (SMR = 1.93), Barranquitas (SMR = 1.88), and Naranjito (1.73), as well as in island municipalities such as Culebra (SMR = 5.12) and Vieques (SMR = 1.49). Figure 2 shows that medium- and high-SVI counties tend to cluster in the central region and some coastal and island municipalities. Mapping of both SVI indexes and SMRs enables a more contextualized interpretation of how social vulnerability may spatially align with suicide risk, and can highlight areas where resource allocation or intervention may be most needed. When comparing maps, there is substantial geographic overlap between counties classified as medium or high SVI and those with elevated SMRs. For example, several of the central counties with the highest SMRs (e.g., Orocovis, Barranquitas, Naranjito) are also categorized as medium or high SVI, suggesting more rural and counties in the central region may face a dual burden of higher social vulnerability and suicide mortality.

It should be noted that while spatial clustering is observed, the choropleth maps in Figs. 2 and 3 do not imply direct statistical associations between CDC-SVI and suicide mortality. However, these visualizations provide important context for identifying regions where prevention efforts or resource allocation may be particularly warranted. Similar patterns were observed when using the SVI-10 (See Supplementary Fig. 2, Additional File 2), albeit slight differences in SVI tertiles categorization in some counties (Supplementary Table 3, Additional File 2).

### Multilevel modeling results

**Fig. 4:**
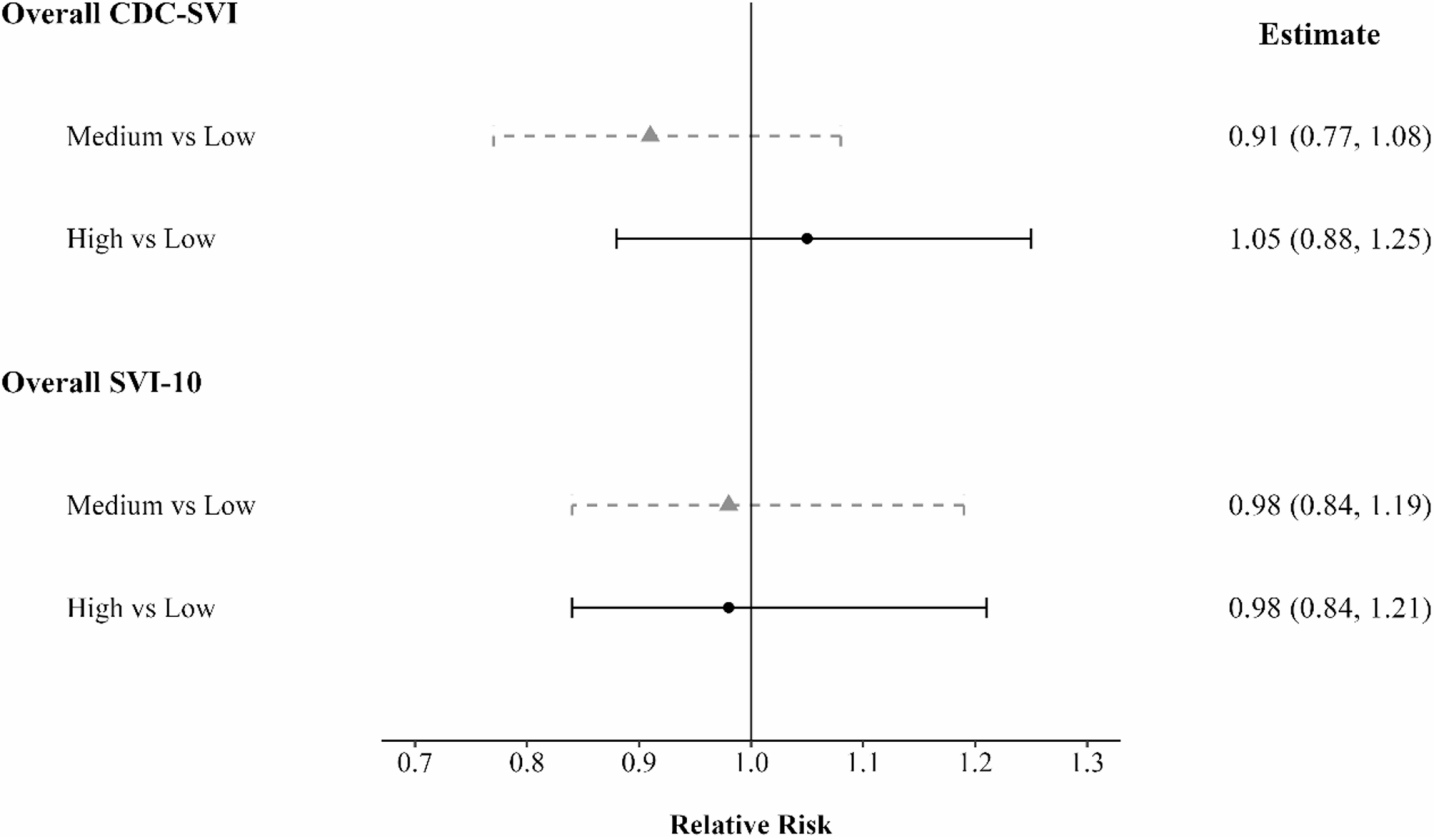
Adjusted relative risks of suicide by county-level social vulnerability tertile: comparison of CDC-SVI and SVI-10 in Puerto Rico, 2017–2022. Relative risks and 95% credible intervals (CIs) were estimated using Bayesian Poisson spatial models (BYM2 specification). Models were adjusted for age, sex, and year. Dots represent posterior means, while horizontal lines represent 95% CIs. Results are shown separately for the CDC-SVI and the SVI-10. Dashed triangles represent estimates for medium-SVI tertiles, and solid circles represent high vs. low comparisons

Figure 4 summarizes results from the adjusted Bayesian Poisson spatial models using INLA. Overall, neither the overall CDC-SVI nor SVI-10 were significantly associated with suicide mortality. In the main Bayesian spatial model (BYM2), relative risk (RR) for suicide deaths comparing counties in the medium-SVI tertile with those in low-SVI tertile was 0.91 (95% CI: 0.77–1.08) for the CDC-SVI and 0.98 (95% CI: 0.84–1.19) for SVI-10. Comparing high-SVI to low-SVI counties, the RR was 1.05 (95% CI: 0.88–1.25) for the CDC-SVI and 0.98 (95% CI: 0.84–1.21) for the SVI-10.

### Sensitivity analyses

Sensitivity analyses supported the robustness of our results across various model specifications. In both the unadjusted models and models with alternative specifications of random effects, results were broadly consistent with the main analysis in that both CDC-SVI and SVI-10 were not statistically associated with suicide mortality rates. Similarly, no significant results were found for theme-specific measures of CDC-SVI and SVI-10. All supplementary analyses and model results are available in Additional File 3, Supplementary Tables 1, 2 and 3.

## Discussion

Using a comprehensive surveillance system of suicide deaths, this study examined the relationship between area-level social vulnerability and suicide mortality across counties in PR using two metrics of community vulnerability to disaster: the CDC-SVI, which is used in the US by state and federal policy-makers for disaster response planning, and the SVI-10, which better reflects the setting of PR. The findings of this study are twofold: First, neither the CDC-SVI nor the SVI-10 were predictive of suicide risk. That is, in contrast to studies in the mainland US that have found that the CDC-SVI is related to mortality risk [64, 65], including suicide mortality [66], we find that suicide mortality did not vary as a function of county-level vulnerability. Second, using the unique data elements of the NVDRS that describe the contributing circumstances to the suicide death, we found that the characteristics, risk factors, and recent life events of suicide decedents in PR also did not vary by area-level social vulnerability. Collectively, these findings call into question whether the operationalization of social vulnerability is able to capture the contextual and structural aspects of PR that contribute to suicide mortality in this setting. Sensitivity analyses further supported the robustness of these findings, with results consistent across unadjusted models and models with alternative specifications of random effects.

PR’s population has endured longstanding economic hardship, demographic shifts, and repeated natural disasters that have shaped community vulnerability and resilience to hazards. Existing evidence highlights that PR also experienced inequities in the allocation and timeliness of federal disaster recovery resources. Following Hurricane’s Harvey, Irma, and Maria in 2017, the federal government responded more rapidly and on a larger scale to Texas and Florida compared to PR [67]. The insufficient and delayed response exacerbates health inequities, underscoring the broader context in which vulnerability metrics are applied in PR. Although this study examines variation only within PR, the socioeconomic range of counties in PR is truncated toward higher vulnerability when compared to the continental US. Even counties classified as “low vulnerability” according to either measure of SVI used in this study in PR are socioeconomically disadvantaged relative to most counties across the US. For example, according to the Federal Emergency Management Agency’s National Risk Index, which uses the CDC-SVI, 61 of PR’s 78 counties are classified as having “very high” vulnerability relative to the rest of the US counties [68]. Consequently, counties classified as having “Low SVI” within PR are low only in comparison to other counties in PR, but most would still be considered highly vulnerable if evaluated against counties in the continental US. This truncation in the SVI distribution in PR vs. the mainland US may contribute to the null results reported here, although other studies have found the SVI-10 to be a salient predictor of population outcomes [49].

In addition, the distribution of mental health and contributing circumstances was similar across CDC-SVI tertiles, indicating that these did not differ substantially by county-level social vulnerability. In our study, this null finding does not appear to be attributable to missing data or under-ascertainment of circumstances, as the proportion of decedents with known circumstances did not differ by SVI tertile. The finding that contributing circumstances do not vary by social vulnerability is consistent with a recent study that used the NVDRS to examine spatial clustering of contributing circumstances in the US (including PR); they found that although some contributing circumstances, like trauma exposure and substance use, are highly correlated and co-located geographically in the continental US, this was not seen in PR [59]. Given the high level of social vulnerability of PR, discussed above, this provides further evidence that the area-level factors that shape suicide in PR are different than those in the US.

While our findings suggest no association between SVI and suicide risk, our geospatial analyses revealed a clustering of suicide and vulnerability in central counties and island-municipalities, which are more rural areas, marked by longstanding economic disadvantage, including limited infrastructure and access to mental health services [9, 29, 69]. This convergence may suggest a structural overlay between socioeconomic hardship and suicide risk that is not represented within the SVI metrics. Future research should explore the creation of new PR-tailored metrics to capture localized, meaningful sources of area-level suicide variation.

Finally, it is essential to understand suicide risk in PR within the broader landscape of violent deaths in this setting. Unlike most US jurisdictions, where suicide is more prevalent than homicide, PR has historically experienced higher rates of homicide than suicide [41, 70, 71]. This pattern of competing mortality risks raises questions about how broader structural conditions may shape the overall burden and distribution of violent deaths. For example, structural drivers such as economic precarity, exposure to community violence, limited access to healthcare and mental health services may elevate suicide or homicide risk, although through different mechanisms.

### Strengths and limitations

This study has several strengths. To our knowledge, it represents an important update to the epidemiologic literature on suicide in PR, addressing a gap since the last population-level study using mortality data from 2000 to 2006 [43]. Key strengths include the use and linkage of population-level suicide mortality data with county-level CDC-SVI data; collaboration with local experts to contextualize findings (including co-principal investigators of the PR site of the NVDRS); and a comprehensive analytic strategy that assessed CDC-SVI and SVI-10 measures using multiple spatial modeling approaches. The use of both the CDC-SVI and the SVI-10 allowed us to compare alternative approaches to measuring social vulnerability within the context of PR.

There are also limitations to note. Analyses were conducted at the county level, which may obscure smaller area-level heterogeneity in vulnerability and suicide risk. Counties in PR often encompass areas of both high and low vulnerability, potentially diluting observed associations in aggregate analyses. While Census tracts would offer greater spatial resolution, the NVDRS does not release data at the Census tract level for suicide, which prevents linkage to data sources at that geographical level. Additionally, we relied on 5-year PRCS estimates to ensure more stable population denominators, which limited our ability to calculate annual suicide rates using reliable, year-specific denominators. Future work would benefit from enhanced geocoding and access to small-area mortality denominators. Despite these limitations, our findings offer important evidence for policy and public health practice. The study’s careful operationalization of vulnerability and multilevel modeling approach provides a more nuanced understanding of suicide risk factors that are essential for public health planning.

## Conclusions

This study explored the relationship between social vulnerability and suicide risk in PR, highlighting both the utility and limitations of SVI metrics. While the CDC-SVI provides valuable insights to identify communities that are most vulnerable to the negative impacts of disasters, and has been used to guide federal funding for disaster preparedness and recovery in PR [48], its value in suicide risk assessment in PR is limited. The utility of the CDC-SVI and other metrics for specific outcomes like suicide depends on the local validity and relevance of their component variables. Finally, the current debate over the politicization and public availability of population health data [72] underscores that the use (or absence) of these tools has real policy and economic implications that directly affect resource allocation and health equity. Ensuring that vulnerability assessment tools are contextually relevant is critical for guiding public health strategies and mental health promotion frameworks that resonate and respond to the unique needs of PR’s population, ultimately advancing mental health equity in the archipelago.

## Supplementary Information

Supplementary Material 1

Supplementary Material 2

Supplementary Material 3

## Data Availability

NVDRS data in this study cannot be shared under the restricted access agreement with the Centers for Disease Control and Prevention (CDC). Data could be obtained directly from the CDC National Center for Injury Prevention and Control through an application process: https://www.cdc.gov/nvdrs/about/nvdrs-data-access.html. CDC-SVI data is available for download here: https://www.atsdr.cdc.gov/place-health/php/svi/svi-data-documentation-download.html.

## Abbreviations

ACS: American community survey
BYM2: Besag-York-Mollié type 2
CDC-SVI: Centers for disease control and prevention - social vulnerability index
CIs: Credible intervals
C/ME: Coroner and medical examiner
DIC: Deviance information criterion
EFA: Exploratory factor analysis
FIPS: Federal information processing standards
INLA: Integrated nested laplace approximation
LE: Law enforcement
NVDRS: National violent death reporting system
PR: Puerto Rico
PRCS: Puerto Rico community survey
RR: Relative risk
SD: Standard deviation
SMRs: Suicide mortality ratios
US: United States
WIC: Watanabe-akaike information criteria
