# Supplementary material for: Exploring social, economic, and environmental correlates of suicide in Puerto Rico, 2017–2022: an ecological cross-sectional study
Source: Inj Epidemiol. 2025 Dec 1;13:3. doi: 10.1186/s40621-025-00632-7 (PMC12777042; doi:10.1186/s40621-025-00632-7)
Supplement: Supplementary file 1 — Supplementary Material 1 [file 40621_2025_632_MOESM1_ESM.docx]

**Additional File 1. Formula for Calculating Suicide Mortality Ratios (SMRs)**

Equations used to calculate expected deaths and suicide mortality ratios (SMRs) at the county level in Puerto Rico, 2017–2022. Expected deaths were derived by applying Puerto Rico’s age- and sex-specific suicide rates to the population distribution of each county. The SMR was then calculated as the ratio of observed to expected deaths.

Expected deaths per stratum:

$$E_{ji}= N_{ji}r_{i}^{std}$$

Where $E_{ji}$= Expected deaths in county $j$, stratum (age*sex group) $i$

$N_{ji}$= population of the county $j$ in the stratum $i$

$r_{i}^{std}=\frac{\sum_{j=1}^{J} O_{ji}}{\sum_{j=1}^{J} N_{ji}}$ = Puerto Rico-wide suicide rate in stratum $i$

Total expected deaths in a county

$$E_{j}= \sum_{i=1}^{I} E_{ji}$$

Observed deaths in the county $j$

$$O_{j}= \sum_{i=1}^{I} O_{ji}$$

Suicide Mortality Ratio (SMR)

$${SMR}_{j}=\frac{O_{j}}{E_{j}}$$
