# Supplementary material for: Exploring social, economic, and environmental correlates of suicide in Puerto Rico, 2017–2022: an ecological cross-sectional study
Source: Inj Epidemiol. 2025 Dec 1;13:3. doi: 10.1186/s40621-025-00632-7 (PMC12777042; doi:10.1186/s40621-025-00632-7)
Supplement: Supplementary file 2 — Supplementary Material 2 [file 40621_2025_632_MOESM2_ESM.docx]

**Additional File 2. Supplementary Methods and Tables: SVI-10**

**Supplementary Table 1**. Vulnerability indicators included in the CDC-SVI and SVI-10

**Supplementary Table 2.** Exploratory Factor Analysis of CDC-SVI: Factor Loadings

**Supplementary Figure 1.** Scree plot and parallel analysis for factor retention

**Supplementary Figure 2.** Map of County-level Social Vulnerability Index-10 (SVI-10) tertiles in Puerto Rico, 2018-2022

**Supplementary Table 3.** Tertiles Comparison: SVI-10 by CDC-SVI

**Supplementary Table 1**. Vulnerability indicators included in the CDC-SVI^1^ and SVI-10^2^

| **CDC-SVI** | **SVI-10** |
| --- | --- |
| *Theme 1: Socioeconomic Status* | |
| Percentage of Persons Below 150% Poverty | Percentage of Persons Below 150% Poverty |
| Percentage of Persons Unemployed | Percentage of Persons Unemployed |
| Housing Cost Burden | Housing Cost Burden |
| Percentage of Persons with No High School Diploma | Percentage of Persons with No High School Diploma |
| Percentage of Persons with No Health Insurance | Percentage of Persons with No Health Insurance |
| *Theme 2: Household Composition* | |
| Percentage of Persons Aged 65 & Older | Percentage of Persons Aged 65 & Older |
| Percentage of Personas Aged 17 & Younger | Aged 17 & Younger |
| Percentage of Civilian Noninstitutionalized Population with a Disability | Percentage of Civilian Noninstitutionalized Population with a Disability |
| Percentage of Single-Parent Households with Children under 18 | Single-Parent Households |
| Percentage of Persons (Age 5+) who “speak English less than well” |  |
| *Theme 3: Racial/Ethnic Minority Status* |  |
| Percentage Minority (Hispanic or Latino (of any race); Black and African American, Not Hispanic or Latino; American Indian and Alaska Native, Not Hispanic or Latino; Asian, Not Hispanic or Latino; Native Hawaiian and Other Pacific Islander, Not Hispanic or Latino; Two or More Races, Not Hispanic or Latino; Other Races, Not Hispanic or Latino) | - |
| *Theme 4: Housing Type & Transportation* | |
| Percentage of Housing in Structures with 10 or More Units |  |
| Percentage of Mobile Homes |  |
| Percentage of Occupied Housing Units with More People than Rooms | Percentage of Occupied Housing Units with More People than Rooms |
| Percentage of Households with No Vehicle Access |  |
| Percentage of Persons in Group Quarters |  |

^1^Agency for Toxic Substances and Disease Registry. CDC SVI. 2024. US Centers for Disease Control and Prevention, Agency for Toxic Substances and Disease Registry. CDC SVI Data & Documentation Download. Available from: https://www.atsdr.cdc.gov/place-health/php/svi/svi-data-documentation-download.html

^2^Indicators excluded from the SVI-10 were removed due to low factor loadings, high complexity, or lack of theoretical relevance for the context of Puerto Rico. Theme 3 (*Racial/Ethnic Minority Status)* was excluded entirely, given that nearly all of PR’s population identifies as Hispanic/Latino.

**Supplementary Table 2.** Exploratory Factor Analysis of CDC-SVI: Factor Loadings^1^

| **Variable** | **Factor 1** | **Factor 2** | **Factor 3** | **Factor 4** | **Communality (h2)** | **Uniqueness (u2)** | **Complexity** |
| --- | --- | --- | --- | --- | --- | --- | --- |
| Percentage of Persons Below 150% Poverty | **0.82** | 0.00 | 0.11 | 0.36 | 0.81 | 0.19 | 1.40 |
| Percentage of Persons Unemployed | 0.09 | 0.00 | 0.08 | 0.34 | 0.13 | 0.87 | 1.20 |
| Housing Cost Burden | **-0.66** | -0.08 | -0.08 | 0.29 | 0.53 | 0.47 | 1.40 |
| Percentage of Persons with No High School Diploma | **0.82** | 0.09 | 0.00 | -0.02 | 0.69 | 0.31 | 1.00 |
| Percentage of Persons with No Health Insurance | **-0.43** | 0.01 | **0.60** | -0.09 | 0.56 | 0.44 | 1.90 |
| Percentage of Persons Aged 65 & Older | 0.18 | **0.87** | -0.01 | -0.04 | 0.80 | 0.20 | 1.10 |
| Percentage of Personas Aged 17 & Younger | 0.17 | **-0.80** | 0.17 | 0.35 | 0.82 | 0.18 | 1.60 |
| Percentage of Civilian Noninstitutionalized Population with a Disability | -0.09 | 0.03 | **-0.43** | 0.02 | 0.20 | 0.80 | 1.10 |
| Percentage of Single-Parent Households with Children under 18 | **-0.40** | -0.23 | -0.03 | **0.45** | 0.41 | 0.59 | 2.50 |
| Percentage of Persons (Age 5+) who “speak English less than well” | **0.84** | -0.25 | 0.03 | 0.31 | 0.86 | 0.14 | 1.50 |
| Percentage Minority | 0.02 | -0.28 | -0.18 | 0.40 | 0.27 | 0.73 | 2.30 |
| Percentage of Housing in Structures with 10 or More Units | **-0.58** | **0.54** | 0.06 | 0.21 | 0.67 | 0.33 | 2.30 |
| Percentage of Mobile Homes | 0.00 | 0.03 | 0.07 | -0.21 | 0.05 | 0.95 | 1.20 |
| Percentage of Occupied Housing Units with More People than Rooms | 0.10 | -0.07 | **0.85** | -0.12 | 0.76 | 0.24 | 1.10 |
| Percentage of Households with No Vehicle Access | 0.03 | 0.35 | **0.46** | **0.40** | 0.49 | 0.51 | 2.90 |
| Percentage of Persons in Group Quarters | -0.18 | 0.14 | -0.15 | 0.20 | 0.11 | 0.89 | 3.70 |

^1^Factor loadings were extracted using minimum residual estimation with varimax rotation. Bolded loadings indicate salient values ≥ |0.40|. Communalities (h2), uniqueness (u2), and item complexity are also shown. This table supports Figure S1 (Scree plot for factor retention based on parallel analysis).

**Supplementary Figure 1.** Scree plot and parallel analysis for factor retention^1^


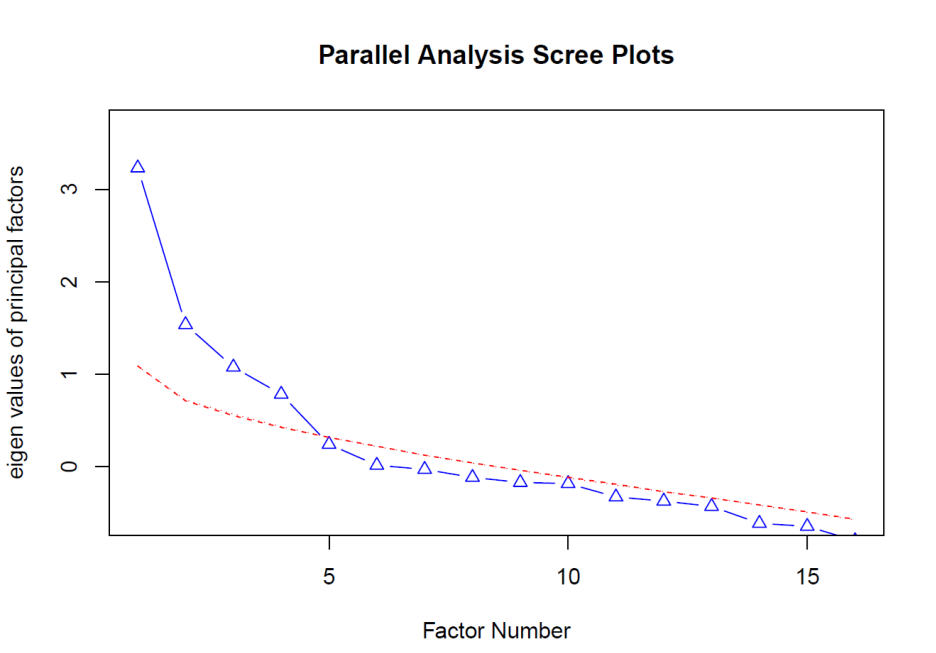


**Figure Legend:** A scree plot with parallel analysis (100 iterations) was used to determine the number of factors to retain. Four factors had eigenvalues exceeding those derived from simulated data, supporting a 4-factor solution for the SVI-10.

**Supplementary Figure 2.** Map of County-level Social Vulnerability Index-10 (SVI-10) tertiles in Puerto Rico, 2018-2022


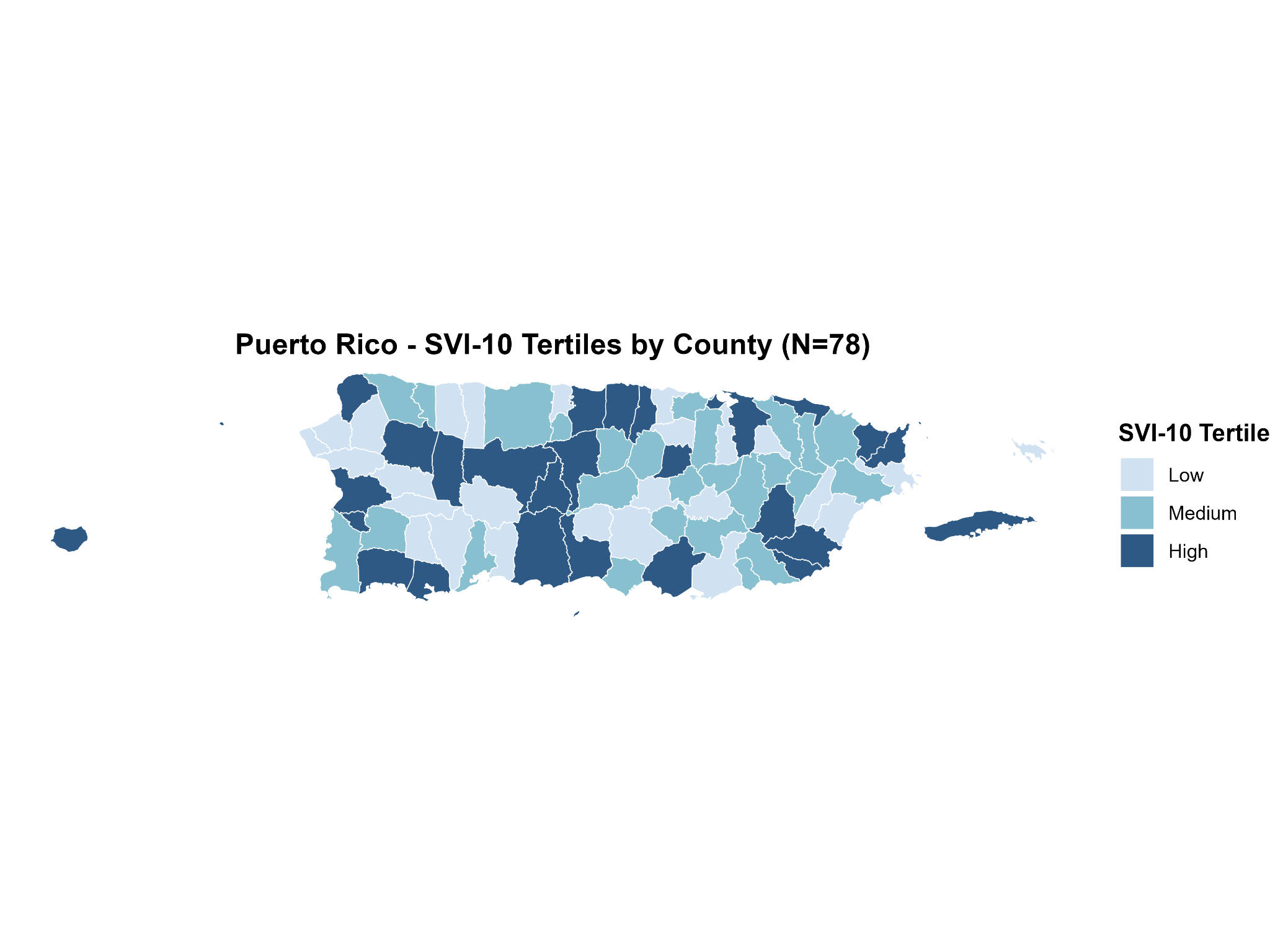


**Figure Legend:** Map of Puerto Rico’s 78 counties shaded by SVI-10 tertile, derived from EFA of CDC-SVI indicators. Lighter shades reflect lower social vulnerability; darker shades reflect higher vulnerability.

**Supplementary Table 3.** Tertiles Comparison: SVI-10 by SVI-16^1^

|  | **CDC-Social Vulnerability Index, N_county_ (col %)** | | |
| --- | --- | --- | --- |
| **Social Vulnerability Index-10** | **Low**  N_county_= 26  N_decedents_= 472 | **Medium** N_county_=26  N_decedents_=454 | **High** N_county_=26 N_decedents_=545 |
| **Low**  N_county_= 26  N_decedents_= 391 | 21 (80.8%) | 4 (15.4%) | 1 (3.8%) |
| **Medium**  N_county_= 26  N_decedents_= 551 | 5 (19.2%) | 18 (69.2%) | 3 (11.5%) |
| **High**  N_county_= 26  N_decedents_= 529 | 0 (0.0%) | 4 (15.4%) | 22 (84.2%) |

^1^ Cross-tabulation of counties and decedents across SVI-10 and CDC-SVI tertiles. Tertiles were assigned at the county level.
