# Supplementary material for: Exploring social, economic, and environmental correlates of suicide in Puerto Rico, 2017–2022: an ecological cross-sectional study
Source: Inj Epidemiol. 2025 Dec 1;13:3. doi: 10.1186/s40621-025-00632-7 (PMC12777042; doi:10.1186/s40621-025-00632-7)
Supplement: Supplementary file 3 — Supplementary Material 3 [file 40621_2025_632_MOESM3_ESM.docx]

**Additional File 3. Supplementary Sensitivity Analyses Results of Bayesian Spatial Models Assessing Social Vulnerability and Suicide Mortality in Puerto Rico**

**Supplementary Table 1**. Results from the unadjusted *BYM2* model and models with alternative specification of structured random effect, using overall CDC-SVI as primary exposure

**Supplementary Table 2.** Results from models with alternative specification of structured random effect, using overall SVI-10 as primary exposure

**Supplementary Table 3.** Results from adjusted BYM21 models with theme-specific CDC-SVI and SVI-10 measures

**Supplementary Tables 1-3** present results from four Bayesian spatial Poisson regression models evaluating the association between county-level social vulnerability (CDC-SVI tertiles & SVI-10) and suicide mortality in Puerto Rico from 2017 to 2022. The outcome is the number of suicide deaths per county, modeled using a log population offset to estimate relative suicide risk. Models were fit using the Integrated Nested Laplace Approximation (INLA) framework. Social vulnerability was operationalized using both CDC-SVI and the SVI-10, categorized into tertiles. All model report relative risks (RR) with 95% credible intervals (CIs). Model fit was assessed using the Deviance Information Criterion (DIC) and Watanabe-Akaike Information Criterion (WAIC), with lower values indicating better fit.

**Supplementary Table 1**. Results from the unadjusted *BYM2* model and models with alternative specification of structured random effect, using overall CDC-SVI as primary exposure

|  | Relative risk (95% Credible Interval) | | | |
| --- | --- | --- | --- | --- |
|  | *Unadjusted BYM2 model*^1^ | *Model with unstructured RE for counties nested within states*^2^ | *Model with structured spatial* RE (Besag CAR)^3^ | *Model with unstructured and structured RE (BYM)*^4^ |
|  | WAIC = 459  DIC = 460 | WAIC = 479  DIC = 474 | WAIC = 464  DIC = 461 | WAIC = 473  DIC = 468 |
| Overall CDC-SVI |  |  |  |  |
| Low | ref | ref | ref | ref |
| Medium | 0.91  (0.77 – 1.08) | 0.90  (0.77–1.05) | 0.93  (0.80–1.09) | 0.92 (0.79–1.06) |
| High | 1.03  (0.87 – 1.22) | 1.04  (0.89–1.20) | 1.07  (0.91–1.26) | 1.05 (0.91–1.22) |
| Population density | - | 1.00  (1.00–1.00) | 1.00  (1.00–1.00) | 1.00 (1.00–1.00) |
| Median distance to nearest emergency department | - | 1.00  (0.96–1.04) | 1.00 (0.96–1.04) | 1.00 (0.96–1.04) |
| Median distance to nearest medical/surgical ICU | - | 1.00  (0.97–1.04) | 1.00 (0.96–1.03) | 1.00 (0.96–1.03) |
| Median distance to nearest hospital with alcohol and drug abuse inpatient care | - | 0.99  (0.99–1.00) | 1.00 (0.98–1.01) | 0.99 (0.99–1.00) |

^1^*Unstructured and structured RE (BYM2):* Includes both unstructured and structured county-level random effects using the reparameterized Besag-York-Mollié 2 (BYM2) model. BYM2 improves upon the traditional BYM model by separating spatially structured and unstructured variation via a mixing parameter, and by applying scaling to ensure the variance is independent of the number of neighbors. No additional covariates are included.

^2^*Unstructured RE for counties nested within states*: Includes only unstructured county-level random effects (IID), adjusting for covariates including population density and healthcare access (median distance to emergency departments, ICUs, and substance use treatment facilities).

^3^*(Structured spatial RE (Besag CAR):* Includes structured spatial random effects based on county adjacency (Besag conditional autoregressive model), adjusting for the same covariates.

^4^*Unstructured and structured RE (BYM):* Includes both unstructured and structured county-level random effects using the original BYM model, adjusted for the same set of covariates.

**Supplementary Table 2**. Results from models with alternative specification of structured random effect, using overall SVI-10 as primary exposure

|  | Relative risk (95% Credible Interval) | | | | |
| --- | --- | --- | --- | --- | --- |
|  | *Unadjusted BYM2 model* | *Adjusted BYM2 model* | *Model with unstructured RE for counties nested within states* | *Model with structured spatial*  RE (Besag CAR) | *Model with unstructured and structured RE (BYM)* |
|  | WAIC = 461  DIC = 461 | WAIC = 464  DIC = 465 | WAIC = 479  DIC = 472 | WAIC = 466  DIC = 463 | WAIC = 480  DIC = 475 |
| Overall SVI-10 |  |  |  |  |  |
| Low | ref | ref | ref | ref | ref |
| Medium | 0.96  (0.81–1.13) | 0.98  (0.84–1.19) | 0.97  (0.83–1.15) | 1.02  (0.86–1.21) | 1.00  (0.86–1.16) |
| High | 0.98  (0.82–1.18) | 0.98  (0.84–1.21) | 0.95  (0.81–1.13) | 1.00  (0.84–1.19) | 0.96  (0.83–1.11) |
| Population density | - | 1.00  (1.00–1.00) | 1.00  (1.00–1.00) | 1.00  (1.00–1.00) | 1.00  (1.00–1.00) |
| Median distance to emergency department | - | 1.00  (0.96–1.05) | 1.00  (0.96–1.04) | 0.99  (0.95–1.04) | 0.99  (0.95–1.03) |
| Median distance to nearest medical/surgical ICU | - | 1.00  (0.96–1.04) | 1.01  (0.97–1.04) | 1.00  (0.96–1.04) | 1.00  (0.97–1.04) |
| Median distance to nearest hospital with alcohol and drug abuse inpatient care | - | 1.00  (0.99–1.01) | 0.99  (0.99–1.00) | 1.00  (0.98–1.01) | 0.99  (0.99–1.00) |

^1^*Unstructured and structured RE (BYM2):* Includes both unstructured and structured county-level random effects using the reparameterized Besag-York-Mollié 2 (BYM2) model. BYM2 improves upon the traditional BYM model by separating spatially structured and unstructured variation via a mixing parameter, and by applying scaling to ensure the variance is independent of the number of neighbors. No additional covariates are included.

^2^*Unstructured RE for counties nested within states*: Includes only unstructured county-level random effects (IID), adjusting for covariates including population density and healthcare access (median distance to emergency departments, ICUs, and substance use treatment facilities).

^3^*(Structured spatial RE (Besag CAR):* Includes structured spatial random effects based on county adjacency (Besag conditional autoregressive model), adjusting for the same covariates.

^4^*Unstructured and structured RE (BYM):* Includes both unstructured and structured county-level random effects using the original BYM model, adjusted for the same set of covariates.

**Supplementary Table 3**. Results from adjusted *BYM2*^1^ models with theme-specific CDC-SVI and SVI-10 measures

|  | Relative risk (95% Credible Interval) | |
| --- | --- | --- |
|  | *CDC-SVI* | *SVI-10* |
|  | WAIC = 472  DIC = 471 | WAIC = 473  DIC = 472 |
| *Theme 1: Socioeconomic Status* | | |
| Low | ref | ref |
| Medium | 1.01 (0.84–1.22) | 1.05 (0.81–1.37) |
| High | 0.91 (0.73–1.14) | 1.01 (0.70–1.44) |
| *Theme 2: Household Composition* | | |
| Low | ref | ref |
| Medium | 1.09 (0.90–1.32) | 1.10 (0.89–1.36) |
| High | 1.20 (0.98–1.45) | 1.19 (0.89–1.59) |
| *Theme 3: Racial/Ethnic Minority Status*^2^ | | |
| Low | ref | ref |
| Medium | 0.96 (0.80–1.15) | - |
| High | 0.89 (0.69–1.13) | - |
| *Theme 4: Housing Type & Transportation* | |  |
| Low | ref | ref |
| Medium | 1.01 (0.83–1.24) | 0.92 (0.77–1.11) |
| High | 0.97 (0.79–1.20) | 1.05 (0.80–1.37) |
| Population density | 1.00 (1.00–1.00) | 0.94 (0.87–1.03) |
| Median distance to emergency department | 1.00 (0.96–1.05) | 0.97 (0.80–1.17) |
| Median distance to Medical/Surgical ICU | 1.00 (0.96–1.04) | 1.04 (0.87–1.24) |
| Median distance to nearest hospital with alcohol and drug abuse inpatient care | 1.00 (0.99–1.01) | 0.97 (0.87–1.10) |

^1^*Unstructured and structured RE (BYM2):* Includes both unstructured and structured county-level random effects using the reparameterized Besag-York-Mollié 2 (BYM2) model. BYM2 improves upon the traditional BYM model by separating spatially structured and unstructured variation via a mixing parameter, and by applying scaling to ensure the variance is independent of the number of neighbors. No additional covariates are included.

^2^Theme 3 (*Racial & Ethnic Minority Status*) was excluded from the SVI-10, given that nearly all of PR’s population identifies as Hispanic/Latino.
